# Acquisition and generalization of emotional and neural responses to faces associated with negative and positive feedback behaviours

**DOI:** 10.3389/fnins.2024.1399948

**Published:** 2024-08-05

**Authors:** Huiyan Lin, Maximilian Bruchmann, Sebastian Schindler, Thomas Straube

**Affiliations:** ^1^Laboratory for Behavioural and Regional Finance, School of National Finance, Guangdong University of Finance, Guangzhou, China; ^2^Institute of Applied Psychology, Guangdong University of Finance, Guangzhou, China; ^3^Institute of Medical Psychology and Systems Neuroscience, University of Münster, Münster, Germany; ^4^Otto Creutzfeldt Center for Cognitive and Behavioral Neuroscience, University of Münster, Münster, Germany

**Keywords:** social learning, generalization, faces, emotional evaluations, fusiform gyrus

## Abstract

Faces can acquire emotional meaning by learning to associate individuals with specific behaviors. Here, we investigated emotional evaluation and brain activations toward faces of persons who had given negative or positive evaluations to others. Furthermore, we investigated how emotional evaluations and brain activation generalize to perceptually similar faces. Valence ratings indicated learning and generalization effects for both positive and negative faces. Brain activation, measured with functional magnetic resonance imaging (fMRI), showed significantly increased activation in the fusiform gyrus (FG) to negatively associated faces but not positively associated ones. Remarkably, brain activation in FG to faces to which emotional meaning (negative and positive) was successfully generalized was decreased compared to neutral faces. This suggests that the emotional relevance of faces is not simply associated with increased brain activation in visual areas. While, at least for negative conditions, faces paired with negative feedback behavior are related to potentiated brain responses, the opposite is seen for perceptually very similar faces despite generalized emotional responses.

## Introduction

Faces can acquire emotional meaning via social learning. Social learning occurs, for example, when neutral faces (conditional stimulus; CS) acquire positive or negative meaning by pairing them with positive or negative information (unconditional stimulus, UCS; e.g., Gast and Rothermund, 2011; Hütter et al., 2022). The acquisition of social conditioning can be observed in emotional evaluations such as valence ratings (Fiedler and Unkelbach, 2011; Hughes et al., 2019). Social learning might also generalize to novel stimuli and/or situations that share similarities with the CS (Dack et al., 2009; Hütter et al., 2014; Hütter and Tigges, 2019; Stegmann et al., 2020). Regarding feedback-associated faces, in two studies of Verosky and Todorov (2010, 2013), participants were asked to learn the associations between neutral faces and socially emotional feedback and subsequently were presented with morphed faces that were perceptually similar to the previously emotion-associated faces. The generalization findings revealed that morphed faces similar to positive- and negative-feedback-associated faces were rated as more pleasant and unpleasant, respectively, than neutral-feedback-associated faces.

From a neuroscientific perspective, the question arises of how social learning acquisition and generalization influence brain responses to faces. Several functional magnetic resonance imaging (fMRI) studies (Davis et al., 2010; Pejic et al., 2013; Schwarz et al., 2013; Bas-Hoogendam et al., 2020) have investigated whether faces that are associated with socially evaluative information activate brain regions such as the amygdala, a brain region that plays a critical role in emotional learning (Davis, 1994; Maren, 1999; Rodrigues et al., 2004; Likhtik and Johansen, 2019), and the fusiform gyrus (FG), a brain region involved in face processing (Kanwisher, 2000; Kanwisher and Yovel, 2006; Bernstein and Yovel, 2015). Both amygdala and FG activations have been shown to reflect the processing of emotional facial expressions (Costafreda et al., 2008; Sergerie et al., 2008; Fusar-Poli et al., 2009a,b; Sabatinelli et al., 2011; Liu et al., 2021; Qiu et al., 2022). It was found that activations in both regions are mainly due to the intensity of facial expressions, irrespective of their valences (Lin et al., 2016, 2020; Müller-Bardorff et al., 2018).

In fear conditioning studies, activations of the amygdala (Gewirtz and Davis, 1998; Maren, 2001; Kim and Jung, 2006; Maren et al., 2013) and visual areas, including FG (Morris et al., 2001; Morris and Dolan, 2004; Dunsmoor et al., 2007), have been shown. However, amygdalar responses seem to be confined to the initial learning of the CS+ (Gewirtz and Davis, 1998; Maren, 2001; Schafe et al., 2001; Kim and Jung, 2006; Maren et al., 2013). Nevertheless, some studies also found generalization effects in the amygdala (Greenberg et al., 2013; Keiser et al., 2017; Lange et al., 2017; Likhtik and Johansen, 2019; Huggins et al., 2021; Webler et al., 2021). No study reported effects in fusiform gyrus to fear-generalized faces.

Regarding different forms of social conditioning, amygdalar activations were increased for neutral faces whose identities gave positive and negative feedback compared to neutral faces (Davis et al., 2010; Pejic et al., 2013; Schwarz et al., 2013; Bas-Hoogendam et al., 2020). However, Todorov and Engell (2008) reported that amygdalar activations were stronger in response to neutral faces that were negatively evaluated than to positively evaluated faces. In addition, this study also showed a similar pattern of relationships between the activation in the FG and the valence of social evaluation. However, other studies did not report activation of the FG to faces associated with social feedback (Schwarz et al., 2013; Bas-Hoogendam et al., 2020). This is in contrast to electrophysiological findings, where the N170, a face-sensitive electrophysiological component of the event-related potential, is potentiated by the emotional relevance of faces (Schindler et al., 2023), and the N170 is at least partially supposed to depend on activation in the FG (Deffke et al., 2007).

Furthermore, to our knowledge, there are no fMRI studies on the generalization of brain responses to faces in social learning designs, except for the study by FeldmanHall et al. (2018) regarding the generalization of the trustworthiness of faces. In this study, participants played a trust game with partners who exhibited trustworthy, neutral, or untrustworthy behaviours and, subsequently, with new partners whose faces were morphed with one of the three original partners. The finding revealed that amygdalar activations were proportional to the degree to which the stimuli resembled the original untrustworthy partner, suggesting that generalization to faces in social learning might be associated with amygdalar activations. No studies—to the best of our knowledge—have investigated the role of the FG on generalization to faces in social learning designs. Nevertheless, one EEG study employing social fear conditioning of faces (Stegmann et al., 2020) showed strongly reduced visual activation to generalized emotional faces. In this study, activation to the face most similar to the CS+ was inhibited despite increased emotional relevance. The authors related this finding to increased visual tuning of the initially learned face with lateral inhibition of the perceptual similar face. They suggested similar effects might be seen in face-responsive regions, such as the FG, which can only be investigated by intracranial recordings or other neuroimaging techniques, such as fMRI.

In the current study, we investigated emotional evaluations and neural responses regarding learned and perceptually similar faces of identities that were associated with negative or positive feedback behaviours. Face similarity was varied by morphing between faces associated with emotional and neutral feedback information. According to the abovementioned studies, we hypothesized that emotional learning would lead to more extreme valence ratings (i.e., more pleasant and more unpleasant) to the face of the relevant identity, and moreover, the effects would generalize to perceptually similar faces. Regarding neural mechanisms, we expected that social learning effects are associated with increased activations in the FG and the amygdala, above all in the negative condition. For generalization stimuli, we expected increased amygdalar activation, especially for negative stimuli compared to neutral stimuli, but we had no clear hypothesis regarding the FG due to the absence of previous fMRI studies.

## Methods

### Participants

A sample of 28 participants (18 women and 10 men, 18–37 years, M ± SD = 21.43 ± 3.80) were recruited in Münster through advertisement. This sample size could obtain a power of >80% to detect a small to moderate effect size for the effect of facial identity (refers to CS+, CS−, and generalized stimuli; *f* = 0.212) based on the power calculation using G*Power 3.1.7 (Faul et al., 2007). One participant was left-handed, and the others were right-handed, as determined by the Edinburgh Handedness Inventory (Oldfield, 1971). Participants had normal or corrected-to-normal vision and did not report current or recent neurological or mental illness. All the participants gave their informed consent to participate in the study, which was in line with standard ethical guidelines from the Declaration of Helsinki. Informed consent of the participants has been obtained to publish the information/images in an online open-access publication. The study was approved by the ethics committee of the University of Münster (approval number: TS 012016; date of approval: 14/03/2016).

### Stimuli

**Figure 1:**
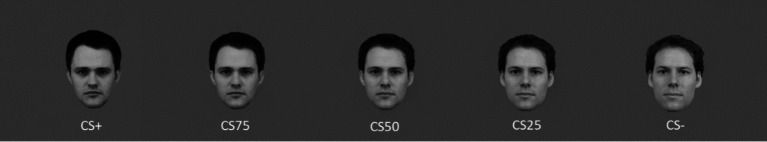
An example of combinations with learned and morphed faces. Facial identities were selected from the Karolinska Directed Emotional Faces (KDEF; Lundqvist et al., 1998) and the Radboud Face Database (Langner et al., 2010).

The stimuli in the present study included facial pictures and a movie. Four male facial identities showing a neutral expression were selected from the Karolinska Directed Emotional Faces (KDEF; Lundqvist et al., 1998) and the Radboud Face Database (Langner et al., 2010). The faces were cropped similarly around the face outline and centered so that the eyes, nose, and mouth were at similar positions. Non-facial parts (e.g., neck, shoulders, and distant hair) were removed. Facial pictures were converted into grey-level images to exclude the influence of hue and colors. The projected images of the faces had a size of about 3.3 × 4.4 degree of visual angle (width × height). We created two stimulus sets consisting of morphed faces constructed from two different pairs of faces using the software PsychoMorph (https://users.aber.ac.uk/bpt/jpsychomorph/; Tiddeman and Perrett, 2002). Each set consisted of five images, which included the two unaltered images of the two identities and three morphs using a 1/3, 1/1, and 3/1 ratio of the identities. Since in each pair, one of the faces was used as a conditioned stimulus (CS+) and one as a neutral stimulus (CS−), we will refer to the faces as 100, 75, 50, 25, and 0% signifying the proportion to which they resemble the CS+ (see Figure 1). In addition, we also created two facial pictures by artificially including freckles on the two 50% faces. One set of faces was assigned to the negative condition, the other to the positive condition.

The video showed an application interview in which actors portrayed an applicant who was interviewed for a job position in a large company and an application committee consisting of four persons. Two committee members, seated in the middle, conducted and evaluated the interview. The other two on the left and right sides were recorders who did not evaluate the applicants. During the movie, the evaluators asked several questions, and the applicant answered these questions. All actors’ faces were masked to reduce the influence on target faces. In addition, the movie had no voices to exclude the vocal effect on the evaluation of target faces. The movie lasted 55 s. The display size of the video was 854 × 480 pixels, and its projected image had a size of about 9.35 × 5.25 degree of visual angle.

### Procedure

**Figure 2:**
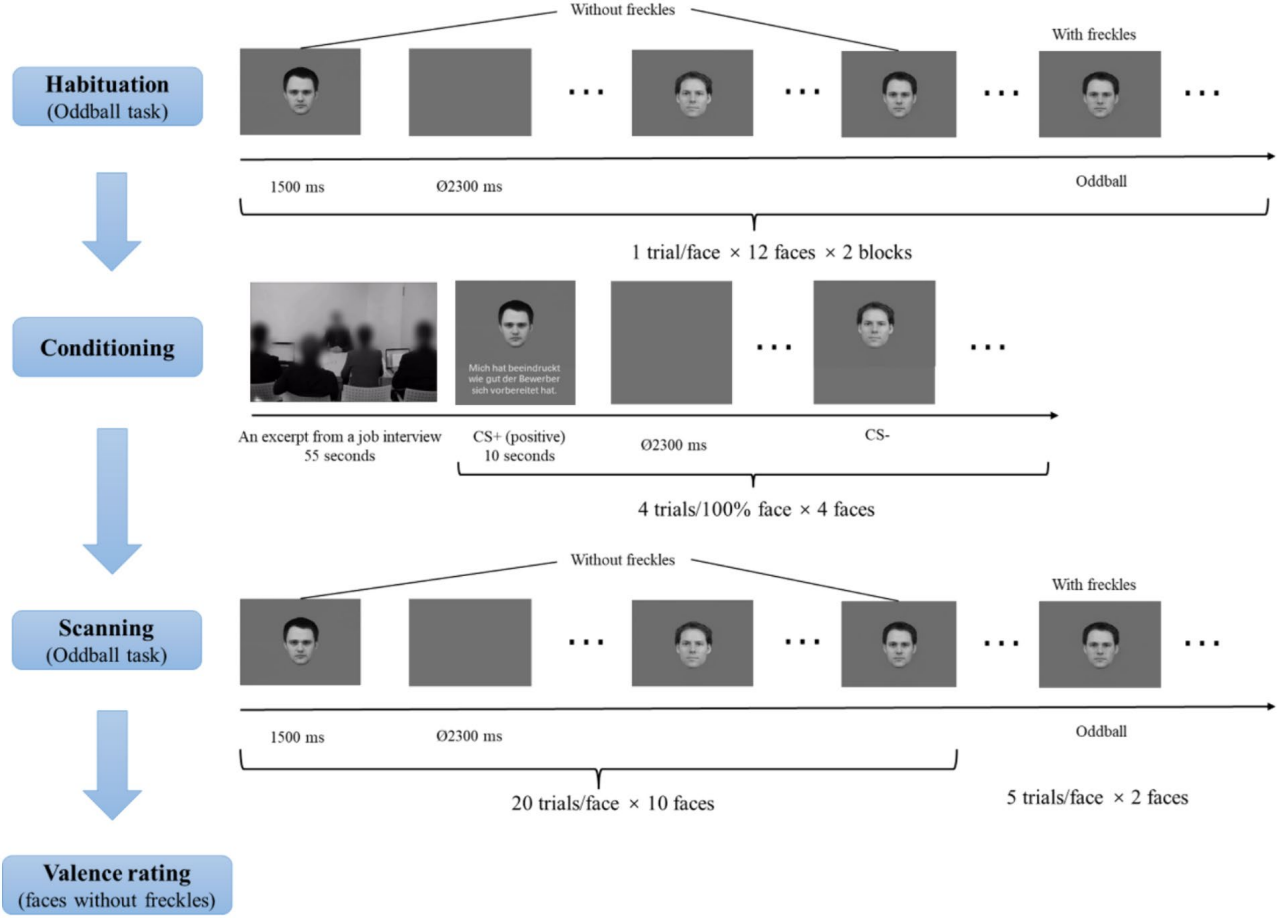
Schematic illustration of the experimental procedure. Facial identities were selected from the Karolinska Directed Emotional Faces (KDEF; Lundqvist et al., 1998) and the Radboud Face Database (Langner et al., 2010).

As shown in Figure 2, participants were first asked to view facial pictures mentioned in the Stimuli section (including faces, morphed faces, and morphed faces with freckles) to habituate to the stimuli. There were two habituation blocks. Per block, each facial picture was presented once for 1,500 ms, with an inter-trial interval (ITI) of 2,300 ms. Participants were told to perform an oddball task, in which they had to indicate the faces with freckles by pressing the left button with the index finger of the right hand using a fiber optic response box (LUMItoucch; Photon Control). These manipulations were used to familiarize participants with the oddball task.

Subsequently, there was a conditioning phase. In this phase, participants saw the video (see the Stimuli section for details) regarding an excerpt from a job interview. Afterwards, participants were presented with a face on the upper side of the screen paired with an evaluative sentence (see the evaluative sentences in Table 1) or no sentences on the lower side of the screen. The faces were the four 100% faces, and two were introduced as the evaluators and the other two as the neutral recorders. The two faces described to be the evaluators served as CS+ faces. One CS+ face was paired with 4 positive evaluative sentences on the applicant, and the other CS+ face was paired with 4 negative evaluative sentences, representing four learning trials per valence. The two faces described as the neutral recorders severed as CS− faces and were not paired with evaluative sentences. Each facial stimulus was presented four times. The assignments of these CS+ and CS− faces were counterbalanced across participants, and the order of the different conditions was randomized. Each facial picture (and the relevant evaluative sentence) was presented for 10 s, with an ITI of 2,300 ms.

**Table 1:** Positive and negative evaluative sentences during face learning.

Valence	Sentences
Positive	*Mich hat beeindruckt, wie gut der Bewerber sich vorbereitet hat*. (I was impressed by how well the applicant prepared.)
	*Ich finde es sympathisch und überhaupt nicht schlimm, dass er die letzte Frage nicht beantworten konnte*. (I think it is likeable and not bad at all that he could not answer the last question.)
	*Ich finde es beeindruckend, wie kompetent er fast alle Fragen beantworten konnte*. (I find it impressive how competently he was able to answer almost all questions.)
	*Dieser authentische und lebendige Bewerber passt genau in unser Team*. (This authentic and lively applicant fits perfectly into our team.)
Negative	*Ich finde es richtig peinlich, dass der Bewerber so nervös war*. (I find it really embarrassing that the applicant was so nervous.)
	*Auf mich macht der Bewerber einen dummen Eindruck, ich würde ihn nicht einstellen*. (The applicant makes a stupid impression on me, and I would not hire him.)
	*Ich finde es richtig peinlich, dass der Bewerber solche Schweißflecke unter den Armen hatte*. (I find it really embarrassing that the applicant had such sweat stains under his arms.)
	*Dass er die letzte Frage nicht beantworten konnte, zeigt doch, was für ein Versager er ist*. (The fact that he could not answer the last question shows what a failure he is.)

During scanning, participants were presented with all facial pictures (including CS+ and CS− faces as well as morphed faces) across two runs. The order of picture presentations was randomized. Each picture was shown once for 1,500 ms, with an ITI between 2,300 ms and 9,200 ms (M = 5,750 ms), optimized by the Optseq algorithm (https://surfer.nmr.mgh.harvard.edu/optseq/; Dale, 1999; Dale et al., 1999). Each face without freckles was presented 20 times, and the two faces with freckles were presented 5 times, resulting in a total of 210 trials with 105 trials per run. Participants were asked to detect faces with freckles.

After the scanning, participants were required to rate the valence of all faces except the oddball stimuli (“1” = “very unpleasant,” “5” = “neutral,” and “9” = “very pleasant”) using a 9-point Likert scale. Stimuli were presented via a back-projection monitor. Stimuli were controlled and behavioral data were recorded using the Presentation software (version 22.1; Neurobehavioral Systems; www.neurobs.de).

### Behavioral data recordings and analyses

For each participant, we averaged the valence ratings. The mean ratings were separately analyzed with repeated measures analyses of variances (ANOVAs) with a within-subject factor facial identity (CS+ versus CS75 versus CS50 versus CS25 versus CS−) per valence condition, followed by post-hoc *t*-tests. Additionally, to understand whether participants paid attention to the stimuli, we also calculated mean hit rates and reaction times and their SEs for with-freckle faces and false alarm rates for without-freckel faces separately in each run of the oddball task during the scanning. Statistical analyses were performed using IBM SPSS Statistics software (Version 28; SPSS INC., an IBM company, Chicago, Illinois). Greenhouse–Geisser corrections were applied to correct degrees of freedom and *p* values and Bonferroni correction was used to correct *post hoc t*-tests when appropriate. A probability level of *p* < 0.05 was considered to be statistically significant.

### FMRI data acquisition and analyses

Structural and functional data were obtained using a 3 Tesla magnetic resonance scanner (“Magnetom Skyra,” Siemens, Medical Solutions, Erlangen, Gemany) with a head coil gradient set. During the tasks, BOLD contrast functional images were acquired using a T2*-weighted echo-planar pulse sequence (TR = 2,300 ms, TE = 30 ms, flip angle = 90°, field of view = 216, matrix size = 64 × 64). For each participant, two runs with 337 volumes per run were acquired. Each volume comprised 42 interleaved axial slices (thickness = 3 mm, gap = 0.3 mm, in-plane resolution = 3 × 3 mm) orientated in an approximately 30° tilted angle from the anterior–posterior commissure plane (Deichmann et al., 2003). The first 5 volumes of each functional run were discarded from analysis to ensure that steady-state tissue magnetization was reached. For anatomical reference, a whole brain high-resolution T1-weighted volume was recorded for each participant during the same experimental session using a 3D spoiled gradient echo pulse sequence.

Functional MRI-data preprocessing and analyses were conducted using the software package BrainVoyager QX (Version 3.6.2; Brain Innovation, Maastricht, The Netherlands). Primarily, all volumes were realigned to the first volume in order to minimize artifacts due to head movements, and a slice time correction was conducted. Further data preprocessing comprised spatial (8 mm full-width half-maximum isotropic Gaussian kernel) as well as temporal smoothing (high pass filter: 10 cycles per run, low pass filter: 2.8 s). The anatomical and functional images were co-registered and normalized to the Talairach space (Talairach and Tournoux, 1988).

Statistical analysis was performed by multiple linear regression of the signal time course at each voxel. The expected BOLD signal change for each event type (predictor) was modeled by a hemodynamic response function. Firstly, voxel-wise statistical maps were generated, and predictor estimates were computed for each individual. The present study included 10 predictors [negative: CS+, CS75, CS50, CS25 and CS−; positive: CS+, CS75, CS50, CS25 and CS−]. The ten movement parameters were modeled as predictors of no interest. Predictor estimates based on voxel-wise statistical maps for each participant were calculated. Fixed-effects single participant level contrast images for planned comparisons of predictor estimates (beta weights) were entered into group-level *t*-tests for a random effect analysis. The present study focused on the bilateral amygdalae and the FG. Thus, data analyses were conducted as regions of interest (ROIs) analysis for these regions. The ROIs for these brain regions were defined based on the automated anatomical atlas (AAL; Rolls et al., 2020). In addition, a whole-brain analysis was performed without *a-priori*-defined ROIs. MNI-coordinates for the ROIs were transformed into Talairach space with ICBM2tal (Lancaster et al., 2007). The FG ROI included all FG-labeled anatomical voxels between −65 and −40 for the *y*-axis according to typical face-related findings in our previous studies (Lin et al., 2016, 2020; Müller-Bardorff et al., 2018; Dellert et al., 2021).

Significant clusters were obtained through cluster-based permutation (CBP) with 1,000 permutations. The non-parametric CBP framework was selected to gain precise false discovery rates without any need for assumptions concerning test-statistic distributions. We separately investigated the differences between the learning (i.e., CS+ vs. CS−) and generalization (i.e., CS75 vs. CS−) effects for the positive and negative conditions. The voxel-level threshold was set to *p* < 0.005. For each permutation, individual beta maps representing activation patterns in a specific effect were randomly assigned without replacement to either of the two groups. The cluster’s mass was assessed by summing all *t*-values in neighboring significant voxels. Subsequently, the observed cluster mass was compared with the distribution of the maximum cluster mass observed in each of the 1,000 permutations. Clusters masses larger than 95% of the permutation distribution were considered statistically significant.

## Results

### Valence ratings

**Figure 3:**
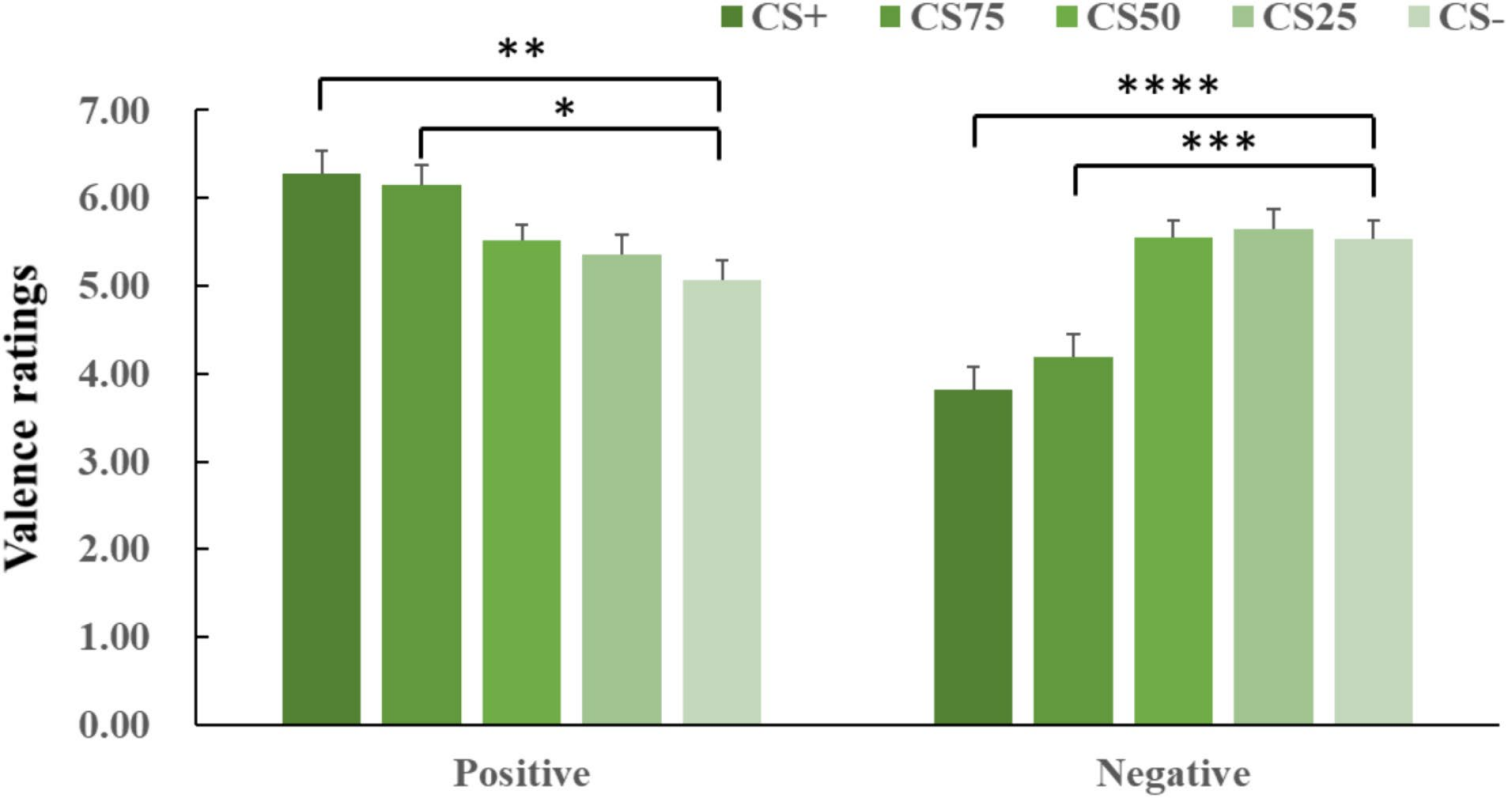
Means and SEs for valence ratings in each experimental condition. The “****,” “***,” “**”, and “*” symbols mean *p*_corrected_ < 0.001, 0.005, 0.01, and 0.05, respectively.

ANOVA for both negative and positive conditions showed an effect of facial identity (negative: *F*(2, 44) = 19.85, *p* < 0.001, $\eta_{p}^{2}$ = 0.424; positive: *F*(2, 63) = 8.25, *p* < 0.001, $\eta_{p}^{2}$ = 0.234). *Post hoc t*-tests for both negative and positive conditions revealed that only CS+ and CS75 faces had more extreme valence ratings (i.e., more unpleasant and more pleasant) than CS− faces (negative: CS+ vs. CS−: *p* < 0.001, Cohen’s *d* = 0.89; CS75 vs. CS−: *p* = 0.004, Cohen’s *d* = 0.77; positive: CS+ vs. CS−: *p* = 0.008, Cohen’s *d* = 0.71; CS75 vs. CS−: *p* = 0.011, Cohen’s *d* = 0.69; Figure 3).

### Performance data for the oddball task

For the faces with freckles, mean hit rates and their SEs were 0.94 ± 0.04 and 0.90 ± 0.05 for the first and second run of the oddball task, respectively, and mean reaction times were 797.46 ± 39.78 ms and 789.61 ± 47.28 ms. Regarding without-freckel faces, mean false alarm rates and their SEs were 0.01 ± 0.01 for both runs. The excellent behavioural performance suggests that participants were hightly attentive to the facial stimuli during the oddball task.

### FMRI results

#### ROI analysis

##### Amygdala

The learning and generalization contrasts for either the positive or negative condition did not show significant activations in the amygdala. An additional analysis that tested possible habituation effects and compared the first vs. second half of the experiment did also not reveal significant effects. However, we would like to note that amygdala activations were apparent on an uncorrected voxel threshold of *p* < 0.05 across negative and positive conditions (CS+ > CS−; left: *x* = −23, *y* = −5, *z* = −3; *t*_max_ = 3.83, cluster size = 7,128 cm^3^).

##### Fusiform gyrus

**Figure 4:**
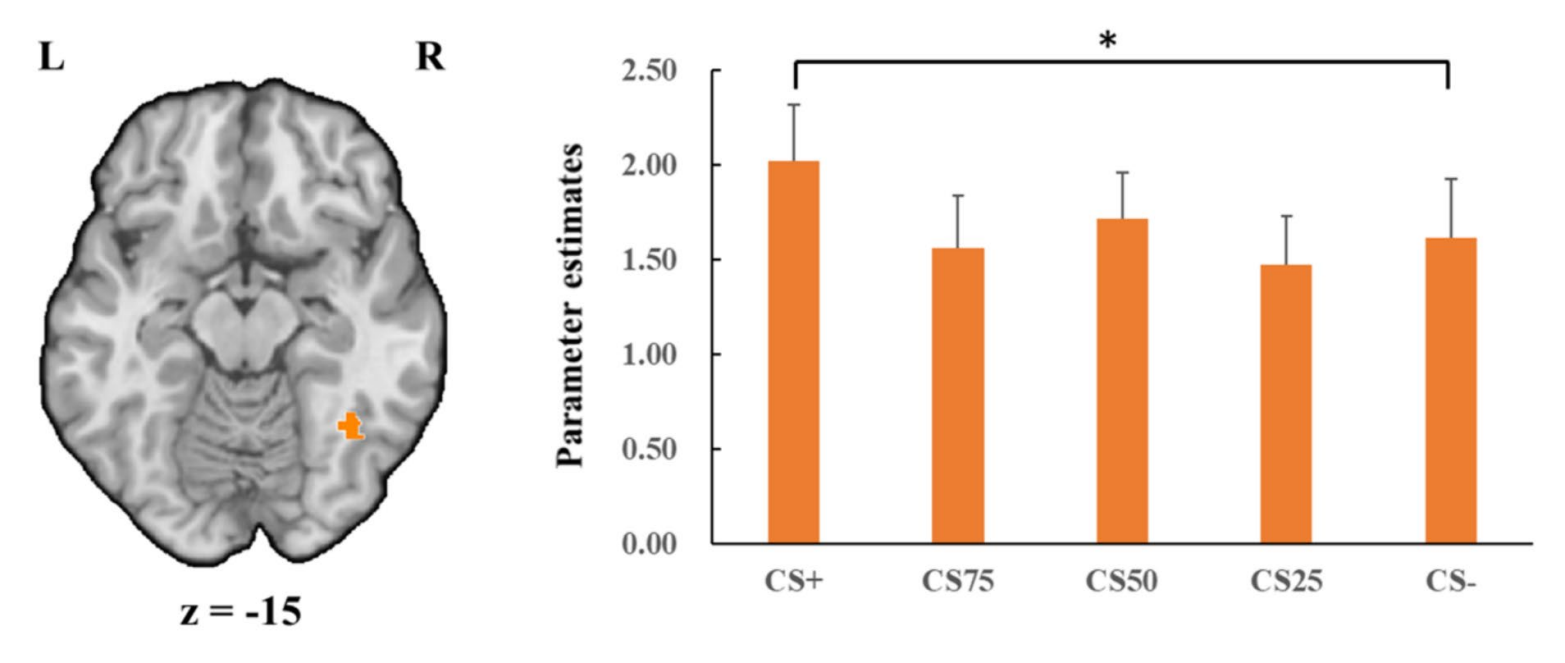
Enhanced activation in right fusiform gyrus (orange) with CS+ faces compared to CS− faces in the negative condition. Bar plots show mean beta values and their SEs for these faces. “*”*p*_corrected_ < 0.05.

**Figure 5:**
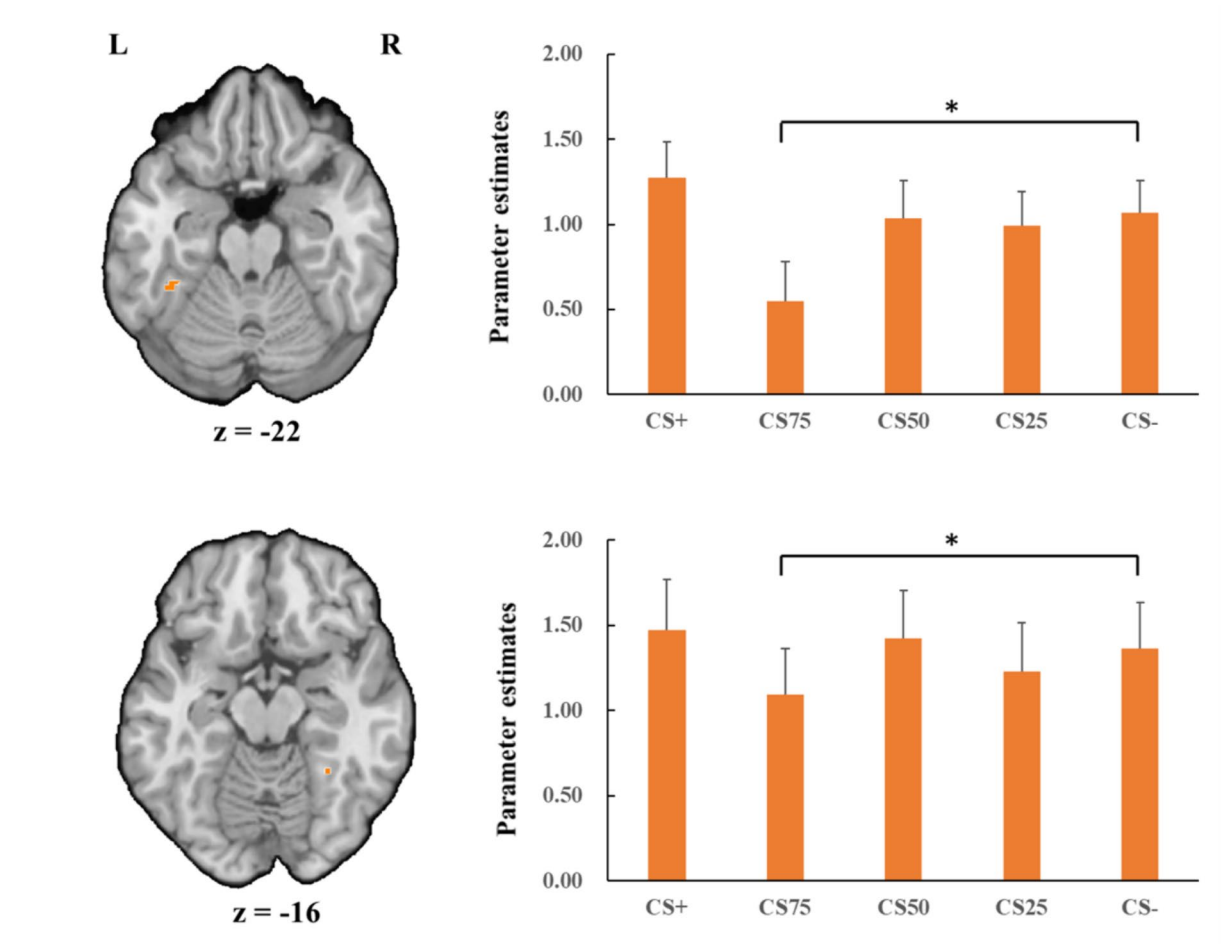
Decreased activation in left and right fusiform gyrus (orange; the upper and lower panel, respectively) with CS75 faces compared to CS− faces in the negative condition. Bar plots show mean beta values and their SEs for these faces. “*”*p*_corrected_ < 0.05.

Concerning the negative condition, the learning contrast revealed a significant cluster in the right FG, with higher activations for CS+ faces than for CS− faces (*x* = 43, *y* = −59, *z* = −15; *t*_max_ = 4.24, *p* < 0.05, CBP corrected; cluster size = 4,293 mm^3^, Figure 4). The reversed learning (CS+ < CS−) contrast showed no significant results. The generalization contrasts revealed significant activation clusters within the left and right FG, with decreased activation for CS75 faces compared to CS− faces (left: *x* = −42, *y* = −44, *z* = −24; *t*_max_ = 3.40, *p* < 0.05, CBP corrected; cluster size = 2,808 cm^3^; right: *x* = 31, *y* = −50, *z* = −18; *t*_max_ = 2.89, *p* < 0.05, CBP corrected; cluster size = 864 mm^3^, Figure 5). There were no significantly increased responses to the CS75 as compared to the CS−.

**Figure 6:**
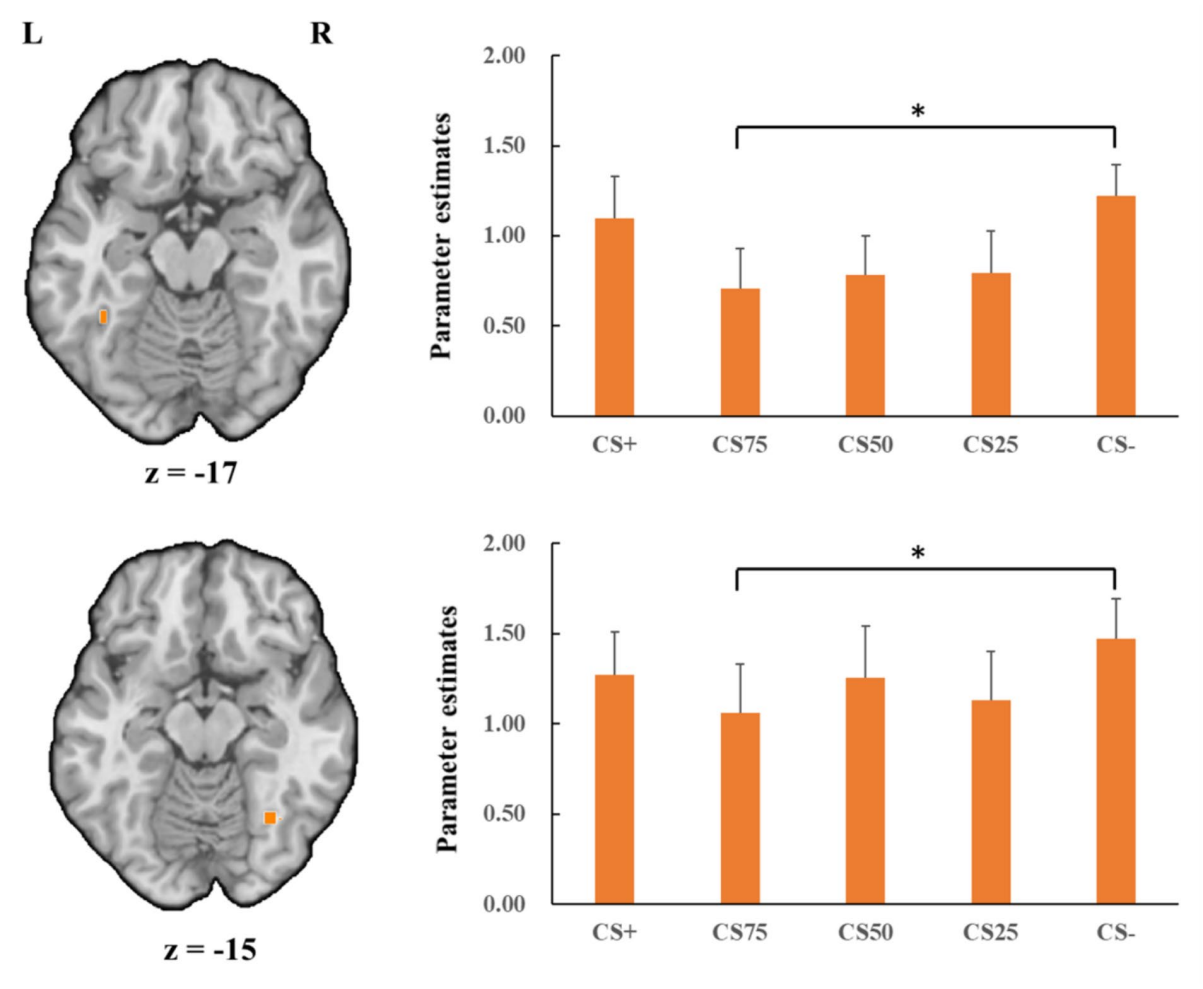
Decreased activation in left and right fusiform gyrus (orange; the upper and lower panel, respectively) with CS75 faces compared to CS− faces in the positive condition. Bar plots show mean beta values and their SEs for these faces. “*”*p*_corrected_ < 0.05.

Regarding the positive condition, there were no effects for the learning contrasts (CS+ vs. CS−). For the generalization contrasts, there were significant clusters in both left and right FG, with decreased activations for CS75 faces than for CS− faces (left: *x* = −38, *y* = −50, *z* = −18; *t*_max_ = 3.16, *p* < 0.05, CBP corrected; cluster size = 3,213 mm^3^; right: *x* = 34, *y* = −65, *z* = −15; *t*_max_ = 3.98, *p* < 0.05, CBP corrected; cluster size = 11,718 mm^3^, Figure 6). We found no significantly increased responses to the CS75 as compared to the CS−.

#### Whole brain analysis

There were a number of brain regions showing increased responses (CS+ > CS−) in the negative condition and increased and decreased responses in the positive condition for the learning contrast, and increased and decreased responses in both the positive and negative conditions for the generalization contrast (see Table 2).

**Table 2:** Significant activations for the (reversed) learning and generalization contrasts.

Regions	Laterality	Talairach coordinates of peak voxel			mm^3^	*t*_(max)_
		*x*	*y*	*z*		
The learning contrast						
Negative						
CS+ > CS−						
Postcentral gyrus	R	55	−17	27	8,019	3.36
	R	52	−18	40	7,290	3.39
Middle occipital gyrus	R	38	−86	5	10,935	3.97
Cerebellum	L	−36	−75	−35	29,160	3.64
CS+ < CS−						
No significant effects						
Positive						
CS+ > CS−						
Postcentral gyrus	R	45	−28	47	10,935	3.73
Middle frontal gyrus	R	25	42	−6	8,019	3.26
CS+ < CS−						
Superior parietal lobule	R	36	−61	50	8,019	4.19
Posterior lobe	R	31	−70	−23	21,870	3.93
Cerebellum	R	13	−85	−30	17,496	3.85
	L	−1	5	16	21,870	5.83
The generalization contrast						
Negative						
CS75 > CS−						
Middle temporal gyrus	L	−51	−71	17	10,206	3.45
CS75 < CS−						
Sub-gyral	R	20	−62	24	17,496	4.00
Parahippocampal gyrus	R	12	−7	−16	7,290	3.49
Lingual gyrus	L	−14	−89	−3	7,290	3.21
Positive						
CS75 > CS−						
Precentral gyrus	R	64	14	9	29,160	5.24
Postcentral gyrus	R	63	−21	29	12,393	5.35
	R	65	−18	17	7,290	3.57
	R	48	−26	42	32,805	3.99
Cerebellum	R	28	−32	−28	8,019	3.57
	L	−18	−44	−27	21,141	3.70
CS75 < CS−						
Sub-gyral	R	36	−73	11	20,412	3.89
Lingual gyrus	R	28	−76	−6	8,748	3.35
Inferior occipital gyrus	L	−38	−86	−5	20,412	3.80

## Discussion

The present study investigated whether positive and negative behaviours of a person influenced emotional evaluations and corresponding neural responses to faces of this person and moreover, whether this effect could generalize to other perceptually similar faces. Behavioural results revealed that participants rated the CS+ and the most perceptually similar face as compared to the CS− faces more negatively in the negative condition and more positively in the positive condition, implying that emotional behaviours of a facial identity influence emotional evaluations of the faces and even of other perceptually similar faces. FMRI results showed increased FG activations to negatively associated faces. More importantly, brain activations in FG to the perceptually most similar face decreased compared to CS− faces for both the negative and positive learning conditions. These findings suggest that social learning of faces is not simply related to increased brain activation in visual areas but might also result in decreased activation in response to a perceptually similar face stimulus.

Regarding emotional evaluations of the valence for faces associated with positive, negative or neutral feedback behaviour, the learning effect is in accordance with previous studies (Verosky and Todorov, 2010, 2013; Pejic et al., 2013; Junghöfer et al., 2017; Wiggert et al., 2017; Hughes et al., 2019; Krasowski et al., 2021; McCrackin et al., 2021; Schindler et al., 2021, 2023). In these studies, neutral faces are perceived as more emotionally relevant when receiving, providing or even merely paired with social feedback. Both previous and current findings suggest neutral faces can acquire emotional evaluations via social learning (Schindler et al., 2021).

Moreover, we also found that the valence ratings of the perceptually most similar faces showed a significant effect according to the acquired emotional associations of faces, suggesting social learning generalizes at least to mostly similar to emotionally associated faces. The current findings are in line with Verosky and Todorov’s studies (2010, 2013). In these studies, morphed faces similar to learned faces paired with positive and negative feedback were rated as more pleasant and unpleasant, respectively, than the morphed faces similar to the learned faces paired with neutral feedback. Therefore, the previous and current findings might indicate that social learning, which is manipulated by associating a facial identity with behavioral feedback, can generalize to other facial identities based on the similarities of facial features. Previous studies have consistently suggested that social learning could generalize to novel stimuli based on similarities in physical features (e.g., colours and shapes; Moran et al., 2023).

Concerning neural activity, our finding revealed increased activation in FG to associated faces in the negative condition. The FG is involved in facial identity recognition (e.g., feature features and configuration; Calder and Young, 2005; Ghuman et al., 2014; Volfart et al., 2022). Previous studies have suggested the role of the FG in face-related fear conditioning (Morris et al., 2001; Morris and Dolan, 2004; Dunsmoor et al., 2007). Moreover, it was found that activations in FG were stronger to neutral faces associated with negative evaluations (Todorov and Engell, 2008). Accordingly, the current finding might suggest that social learning strengthens identity recognition of negatively associated faces.

While the current finding is in line with Todorov and Engell’s study (2008), other studies did not observe such activations (Schwarz et al., 2013; Bas-Hoogendam et al., 2020). In these studies, neutral faces, who gave emotional evaluations to the participants (Bas-Hoogendam et al., 2020) or to both the participants and other persons (i.e., self-related and -unrelated contexts; Schwarz et al., 2013), did not activate FG. Future studies are necessary to reveal the basis for inconsistencies. However, it should be noted that, for electrophysiological studies, it has been suggested that the emotional relevance of faces reliably increases N170 amplitudes when the association of a neutral face with emotional relevance is established (Schindler et al., 2023). Furthermore, N170 effects seem to depend on the strengths of learning the emotional relevance of faces and the intensity of the emotional information (Schindler et al., 2023).

In the present study, we found only significant learning effects in the FG to negative faces. This might suggest a negativity bias in face learning (Righi et al., 2012; Jackson et al., 2014; Stiernströmer et al., 2016). However, we suggest that studies with increasing relevance and intensity of the positive condition might also reveal effects in FG. At least for facial expressions, we have shown that brain responses strongly depend on the intensity of facial expressions regardless of valence (Lin et al., 2016, 2020; Müller-Bardorff et al., 2018).

Remarkably, our current finding also revealed a decreased activation in FG to the successful generalization face as compared to neutrally associated faces, irrespective of valence. This effect is similar to a recent EEG study (Stegmann et al., 2020), which revealed decreased responses of the steady-state visually evoked potential (ssVEP) for faces that were perceptually very similar to the CS+ (neutral faces that were paired with negative voices). This ssVEP is thought to reflect the responses of visuocortical areas. In accordance with suggestions by previous studies (McTeague et al., 2015; Stegmann et al., 2020), decreased visuocortical activity, including decreased FG activation, might be explained by lateral inhibitory interactions between neuronal populations in face-sensitive cortical areas. From an evolutionary perspective, it is adaptive for individuals to enhance sensory specificity in the visual cortex to distinguish the motivational information-providing stimulus (e.g., emotionally associated faces) from other sensory signals (e.g., perceptually similar faces; Miskovic and Keil, 2012). In this case, signals from frontoparietal attention networks may selectively facilitate presentations of emotionally associated faces in the visual cortex by prompting local inhibitory interactions between adjacent cortical units. This process is supposed to prompt suppression of the features represented by the most similar stimuli, even though this similar stimulus is associated with generalized emotional responses (McTeague et al., 2015; Stegmann et al., 2020). Furthermore, the findings might also be associated with the ambiguity of morphed faces and reduced individualization and recognition of these faces (Onat and Büchel, 2015).

Thus, considering all studies, the findings suggest that the generalization of emotional learning decreases the representation of facial identity in face-sensitive visual areas. However, it has to be noted that even fMRI research reflects activation of a large number of neurons, and it remains to be investigated, for example, by using higher spatial resolution with 7 T scanners, whether a sharpening of representations of generalized faces might be observed with increased activations in a very small part of the FG but decreased activations in other parts.

In contrast to our hypothesis and some previous studies (Davis et al., 2010; Pejic et al., 2013; Schwarz et al., 2013; Bas-Hoogendam et al., 2020), we did not find altered amygdalar activations to facial identities that were paired with emotionally evaluative information. It has been suggested that amygdalar activations involving associated learning depend on factors such as stimulus salience and specific experimental designs (Visser et al., 2021). We showed in previous studies that amygdalar activations to facial expressions are associated with emotional intensity of stimuli regardless of the valence of facial expression (Lin et al., 2016, 2020; Müller-Bardorff et al., 2018). For the current study, feedback stimuli (i.e., evaluative sentences) were moderately positive and negative, which might be insufficient to detect amygdalar activations at the required strong statistical thresholds (please note the potential effects on uncorrected thresholds). Furthermore, amygdala activation is associated with habituation gradients (e.g., Büchel et al., 1998; Sperl et al., 2019). In addition, several fear conditioning studies failed to show amygdalar activation after the learning phase (Phelps et al., 2004; Greenberg et al., 2013; Onat and Büchel, 2015; Fullana et al., 2016; Lange et al., 2017; Likhtik and Johansen, 2019; but see Straube et al., 2007; Sperl et al., 2019), which might also be relevant for social conditioning designs, despite the fact that we did not reveal this effect in our study.

Finally, we would like to mention several limitations of the current study and suggest ideas for future investigations. First, we only used a specific form of a social learning design with moderate changes in valence ratings. Other designs might lead to stronger emotional learning effects (Junghöfer et al., 2017; McCrackin et al., 2021). This might be especially relevant for the positive condition. Thus, future studies could use other social learning designs with more intense emotional information of faces regarding associated information and/or strength of learning. Second, our findings were based on a moderate sample size, which was not sufficient to reveal small effects. Future studies might expand the sample size to investigate relevant social learning and generalization effects, particularly the effects of social learning in the positive condition. Moreover, future studies with larger samples could also investigate inter-individual differences depending on specific learning and generalization effects. This would also allow for investigating lateralization effects due to handiness differences, for example, regarding activation in FG (Sha et al., 2021).

## Conclusion

The current study revealed that observing faces providing positive and negative social feedback to others led to more extreme valence ratings for faces associated with negative and positive information and perceptually similar neutral faces. The findings also show increased FG activation to learned faces in the negative condition. More importantly, FG activity to the perceptually most similar faces was decreased compared to CS− faces regardless of valence. These findings suggest that the emotional relevance of faces is not only associated with increased activity in visual areas but also with inhibitory responses to the face, which is most similar to the initially learned facial stimulus.

## Data availability statement

The raw data supporting the conclusions of this article will be made available by the authors, without undue reservation.

## Ethics statement

The studies involving humans were approved by the ethics committee of University of Münster. The studies were conducted in accordance with the local legislation and institutional requirements. The participants provided their written informed consent to participate in this study.

## Author contributions

HL: Formal analysis, Writing – original draft, Writing – review & editing. MB: Formal analysis, Investigation, Methodology, Writing – review & editing. SS: Methodology, Writing – review & editing. TS: Conceptualization, Investigation, Supervision, Writing – review & editing.
